# Givinostat-Liposomes: Anti-Tumor Effect on 2D and 3D Glioblastoma Models and Pharmacokinetics

**DOI:** 10.3390/cancers14122978

**Published:** 2022-06-16

**Authors:** Lorenzo Taiarol, Chiara Bigogno, Silvia Sesana, Marcelo Kravicz, Francesca Viale, Eleonora Pozzi, Laura Monza, Valentina Alda Carozzi, Cristina Meregalli, Silvia Valtorta, Rosa Maria Moresco, Marcus Koch, Federica Barbugian, Laura Russo, Giulio Dondio, Christian Steinkühler, Francesca Re

**Affiliations:** 1 School of Medicine and Surgery, University of Milano-Bicocca, 20854 Monza, Italy; mariasilvia.sesana@unimib.it (S.S.); marcelo.kravicz@unimib.it (M.K.); f.viale1@campus.unimib.it (F.V.); rosa.moresco@unimib.it (R.M.M.); francesca.re1@unimib.it (F.R.); 2 APHAD srl, 20090 Buccinasco, Italy; c.bigogno@aphad.eu (C.B.); g.dondio@aphad.eu (G.D.); 3 Experimental Neurology Unit, School of Medicine and Surgery, University of Milano-Bicocca, 20900 Monza, Italy; eleonora.pozzi@unimib.it (E.P.); laura.monza@unimib.it (L.M.); valentina.carozzi1@unimib.it (V.A.C.); cristina.meregalli@unimib.it (C.M.); 4 Institute of Bioimaging and Molecular Physiology (IBFM), National Research Council (CNR), 20054 Segrate, Italy; silvia.valtorta@ibfm.cnr.it; 5 INM—Leibniz Institute for New Materials, Campus D2 2, 66123 Saarbrücken, Germany; marcus.koch@leibniz-inm.de; 6 Department of Biotechnology and Biosciences, University of Milano-Bicocca, Piazza della Scienza 2, 20126 Milan, Italy; f.barbugian@campus.unimib.it (F.B.); laura.russo@unimib.it (L.R.); 7 CURAM, SFI Research Centre for Medical Devices, National University of Ireland Galway, H91 TK33 Galway, Ireland; 8 Italfarmaco SpA, 20092 Cinisello Balsamo, Italy; c.steinkuhler@italfarmaco.com

**Keywords:** glioblastoma, liposomes, HDAC inhibitor, brain, cancer

## Abstract

**Simple Summary:**

Glioblastoma is the most common malignant brain tumor with a high grade of recurrence, invasiveness, and aggressiveness. Currently, there are no curative treatments; therefore, the discovery of novel molecules with anti-tumor activity or suitable drug delivery systems are important research topics. The aim of the present study was to investigate the anti-tumor activity of Givinostat, a pan-HDAC inhibitor, and to design an appropriate liposomal formulation to improve its pharmacokinetics profile and brain delivery. The present work demonstrates that the incorporation of Givinostat in liposomes composed of cholesterol and sphingomyelin improves its in vivo half-life and increases the amount of drug reaching the brain in a mouse model. Furthermore, this formulation preserves the anti-tumor activity of glioblastoma in 2D and 3D in vitro models. These features make liposome-Givinostat formulations potential candidates for glioblastoma therapy.

**Abstract:**

Glioblastoma is the most common and aggressive brain tumor, associated with poor prognosis and survival, representing a challenging medical issue for neurooncologists. Dysregulation of histone-modifying enzymes (HDACs) is commonly identified in many tumors and has been linked to cancer proliferation, changes in metabolism, and drug resistance. These findings led to the development of HDAC inhibitors, which are limited by their narrow therapeutic index. In this work, we provide the proof of concept for a delivery system that can improve the in vivo half-life and increase the brain delivery of Givinostat, a pan-HDAC inhibitor. Here, 150-nm-sized liposomes composed of cholesterol and sphingomyelin with or without surface decoration with mApoE peptide, inhibited human glioblastoma cell growth in 2D and 3D models by inducing a time- and dose-dependent reduction in cell viability, reduction in the receptors involved in cholesterol metabolism (from −25% to −75% of protein levels), and reduction in HDAC activity (−25% within 30 min). In addition, liposome-Givinostat formulations showed a 2.5-fold increase in the drug half-life in the bloodstream and a 6-fold increase in the amount of drug entering the brain in healthy mice, without any signs of overt toxicity. These features make liposomes loaded with Givinostat valuable as potential candidates for glioblastoma therapy.

## 1. Introduction

Histone acetylation and deacetylation dynamically affect DNA structure, leading to the activation or suppression of gene transcription. These processes are mediated by two families of enzymes: histone acetyltransferases (HATs) and histone deacetylases (HDACs), respectively. Genes whose expression is affected by histone acetylation changes are frequently involved in the control of cell cycle progression, differentiation, and apoptosis [1,2]. HDACs are nuclear and/or cytosolic enzymes divided into four classes based on their homology to yeast proteins: class I, II, and IV HDACs are zinc-dependent hydrolases while class III HDACs, the sirtuins, couple lysine deacetylation to NAD hydrolysis. Epigenetic dysregulation of histone-modifying enzymes is commonly found in many tumors and has been linked to cancer proliferation, changes in metabolism, drug resistance, migration, angiogenesis, and escape from the immune system [3,4]. This theme has inspired researchers to develop different classes of HDAC inhibitors (HDACis), some of which are in clinical trials for the treatment of tumors [5,6]. Among HDACis, Givinostat (ITF2357) is a potent pan-HDAC inhibitor that was first described by Leoni F. et al. in 2005 [7]. It has completed phase II clinical trials for polycythaemia vera [8] and is presently being evaluated in a phase III trial for Duchenne muscular dystrophy. The achievement of this advanced drug development phase was determined by the superior tolerability of Givinostat with respect to other already approved HDACis [9].

Pre-clinical data indicate the potential anti-tumor activity of Givinostat on solid tumors, including brain tumors [10,11,12]. Among them, glioblastoma multiforme (GBM, a grade IV astrocytoma) is the most common malignant brain tumor and is responsible for 46.1% of all primary malignant brain tumors [13]. This tumor displays aberrant expression and/or defective activity of HDACs, which have been linked to tumorigenesis [14]. The current standard care for GBM is based on Stupp’s protocol, which includes radiotherapy and a concomitant treatment with temozolomide (TMZ) chemotherapy after surgical resection of the primary tumor mass [15].

Unfortunately, GBM shows a high grade of recurrence mainly attributable to GBM stem cells (GSCs) [16], which is the reason for the median lifespan from the time of diagnosis to death of approximately 15 months [17]. Since there are no curative treatments for GBM and the prognosis is poor, finding novel molecules with antitumor activity, or developing suitable delivery systems for already existing drugs are important research topics. 

The anti-proliferative and pro-apoptotic efficacy of Givinostat has been demonstrated on GSCs [18]. Together with its ability to revert the transformed phenotype, anti-cancer efficacy has also been shown in in vivo models of GBM [12]. Nevertheless, the ability of Givinostat to cross the blood-brain barrier (BBB) and reach the brain parenchyma at therapeutic doses have never been directly demonstrated but only deduced from the downstream effects of its administration [12]. Moreover, the use of novel drug delivery systems to improve its pharmacokinetics and the therapeutic index and reduce its side effects, such as thrombocytopenia, has not been investigated yet. 

Among the different drug delivery systems available, liposomes are the most used for the transport of a variety of anti-cancer agents directly to tumors, including GBM. Liposomes offer many advantages, including synthetic flexibility, biodegradability, biocompatibility, low immunogenicity, and toxicity. Accordingly, several liposomal formulations have been approved by the United States Food and Drug Administration (FDA). Moreover, ligand attachment to the surface of liposomes has facilitated active targeting and subsequent improved therapeutic efficacy of different chemotherapeutic drugs [19,20,21].

In this context, we evaluated the pharmacokinetics of Givinostat and its metabolites in a healthy animal model. Additionally, we designed an appropriate liposomal formulation to improve the drug half-life in the systemic circulation and enhance its brain delivery. Moreover, the efficacy of Givinostat after incorporation in liposomes was investigated in 2D and 3D in vitro models composed of human GBM cells, and further studies were performed to determine their mechanism of action and potential use for GBM treatment.

## 2. Materials and Methods

### 2.1. Materials

Givinostat (ITF2357; [6-diethylaminomethyl) naphtalen-2-yl] methyl N-[4-(hydroxycarbamoyl) phenyl] carbamate) was synthetized and characterized by Italfarmaco S.p.A. Free Givinostat stock solution was prepared by diluting the powder in DMSO at a concentration of 2 mM and stored at −20 °C until use.

Cholesterol (Chol) was purchased from Sigma-Aldrich (Milano, Italy). 1,2-Distearoyl-sn-glycero-3-phospho-ethanolamine-N[maleimide(polyethyleneglycol)-2000] (mal-PEG-DSPE) and sphingomyelin from bovine brain (Sm) were purchased from Avanti Polar Lipids, Inc (Alabaster, AL, USA).

### 2.2. Animals

Five-week-old healthy Swiss CD1 mice (25–30 g of body weight) were purchased from Envigo (Italy). The animals were housed under a 12-h light/dark cycle in a controlled environment (22 ± 2 °C with a relative humidity of 55 ± 10%) in the institutional animal facility with ad libitum access to food and water. Animal care and husbandry were conducted in conformity with the institutional guidelines in compliance with national (d.lgs. 26/2014, Gazzetta Ufficiale della Repubblica Italiana, n. 61, 14 March 2014) and international laws and policies (European Union directive 2010/63/UE; Guide for the Care and Use of Laboratory Animals, U.S. National Research Council, 1996). The procedures were authorized by the Italian Ministry of Health (Protocol FB7CC.5.EXT.39, 28 September 2021, authorization number 433/2016-PR).

### 2.3. Preparation and Physico-Chemical Characterization of Liposomes

Marqibo^®^-like small unilamellar liposomes were formulated. Liposomes composed of Chol/Sm/mal-PEG-DSPE (48.75/48.75/2.5 molar ratio) [22], combined with 1 mol% BODIPY^TM^-Sm for CLS experiments, were prepared by the extrusion procedure. Briefly, lipids were mixed in CHCl_3_/CH_3_OH (2:1, *v*/*v*) and dried under a gentle stream of nitrogen followed by a vacuum pump for 3 h to remove organic solvent. The resulting lipid film was rehydrated in 62.5 mM sucrose octasulfate-ammonium salt (SOS-AS) solution (pH 4.5) for 1 h at 65 °C, vortexed, and then extruded through 200- and 80-nm polycarbonate membrane filters at 60 ± 4 °C under 20 bar nitrogen pressure. Liposomes were then dialyzed against 10% sucrose (pH 5.5) at RT for 48 h [23] using Dialysis membrane Spectra/Por^®^ 1, 6–8K MWCO (Spectrum Medical Devices, CA). Givinostat was dissolved in 65 °C water at a concentration of 6 mg/mL for 1 h. Drug loading was carried out by adding 2 mg/mL of Givinostat to 20 mM (total lipids) liposomes and the pH was adjusted to 4.0 with HCl. The mixture was incubated at 65 °C for 1 h. Unencapsulated drug was removed by Amicon^®^Ultra 10 kDa Protein Purification and Concentration Filters (Merck, Darmstadt, Germany). The yield of Givinostat encapsulation was calculated by measuring the OD_265nm_ of the unencapsulated drug compared to the total drug loaded into the liposomes preparation using a calibration curve for free Givinostat dissolved in water at 65 °C. Lipid recovery was estimated by Stewart assay [24]. The encapsulation efficiency (EE%) and drug-to-lipid mass ratio (D/L, μg/μg) were calculated as described [25]. After purification, sample solutions were adjusted to pH 7.0 by adding PBS and stored at 4 °C until use. The liposome surface was functionalized with mApoE peptide (CWGLRKLRKRLLR, Karebay Biochem, Monmouth Junction, NJ, USA), exploiting the thiol–maleimide coupling reaction, as described [26]. Unfunctionalized liposome preparations were named LIP-GIV while mApoE-functionalized liposome preparations were named LIP/m-GIV.

The morphology of the liposomes was characterized by cryo-EM as follows: 3 µL of the aqueous solution was placed on a porous carbon supporting TEM grid (Plano, Wetzlar, Germany, type S147-4), blotted for 2 s, and plunged into liquid ethane at −165 °C using a Gatan (Pleasanton, CA, USA) CP3 plunge freezer. The vitrified sample was transferred under liquid nitrogen to a Gatan model 914 cryo-TEM holder. Bright-field TEM imaging was performed at −170 °C and 200 kV accelerating voltage using a JEOL (Tokio, Japan) JEM-2100 LaB_6_ transmission electron microscope equipped with a Gatan Orius SC1000 CCD camera operating under low-dose conditions. Size, polydispersity index (PDI), and ζ-potential were analyzed using the dynamic light scattering (DLS) technique and interferometric Doppler velocimetry (Brookhaven Instruments Corporation, Holtsville, NY, USA equipped with ZetaPALS device) as previously described [26]. Stability was measured by following the size, PDI, and ζ-potential and drug release for three weeks. The amount of drug released from the liposomes was determined by measuring OD_265nm_ of the free Givinostat fraction, collected after sample centrifugation (Amicon^®^Ultra 10 kDa).

### 2.4. Pharmacokinetics and Brain Penetration

Givinostat 7.5 mg/kg, free (dissolved in 5% DMSO and 95% PEG400/H_2_O: 1/1) or encapsulated in liposomes (dissolved in PBS), was administered by intravenous (i.v.) injection into 5-week-old healthy Swiss CD1 mice (n = 72, 3 mice/time point). Mice were sacrificed at different time points up to 48 h after the injection and blood and brain were harvested. Blood was collected from the cava vein in tubes coated with Li-heparin anticoagulant and centrifuged at +4 °C, 3000 g for 10 min to obtain the plasma. After blood collection, brain was harvested, washed in saline, dried on absorbent paper, weighed, and placed into appropriate tubes. All samples were analyzed for their Givinostat content using the LC-MS/MS method.

### 2.5. LC-MS/MS Analysis

Stock solution of Givinostat, ITF2374, ITF2375 (metabolites), and internal standard ITF2400 were prepared in ACN/water 1:1 at 1 mg/mL. Working solutions were prepared by sequential dilution in water:ACN 8:2. Brains were homogenized in 20 mM ammonium formate buffer (1 g/5 mL). In total, 45 µL of blank plasma or brain homogenates was added to 200 µL of ACN containing ITF2400 at 25 ng/mL and acted as internal standards. Samples were vortexed for 3 min and centrifuged for 10 min at 5 °C at 500 rpm. Samples were transferred into a 96-well plate, dried under nitrogen flow, and resuspended in 200 µL 0.1% FA H_2_O/ACN (75:25). After vortexing, samples were injected into LC-MS/MS. Samples were analyzed on a UPLC Acquity (Waters, Milford, MA, USA) coupled with an API 3200 Triple Quadrupole (ABSciex). Mobile phases were water and ACN with 0.1% FA on a Kinetex 2.6 µm C18 100 A 75 × 3 mm (Phenomenex, Torrance, CA, USA). Analytes were quantified in MRM ESI positive mode. MRM transitions for qualification and quantification and MS parameters are reported in Table S1. Representative chromatograms of blank and LLOQ of Givinostat and metabolites in plasma and brain are reported in Figure S1.

The Givinostat analytical ranges were as follows: plasma 2.5–4000 ng/mL; brain 0.5–2000 ng/mL; the ITF2374 analytical ranges were as follows: plasma 0.1–1000 ng/mL and brain homogenate 0.5–500 ng/mL; the ITF2375 analytical ranges were as follows: plasma 0.5–1000 ng/mL and brain homogenate 0.5–500 ng/mL. The calibration curves are reported in Figure S3.

### 2.6. Pharmacokinetics Analysis

Pharmacokinetic parameters were calculated using Excel Add in (PK Solver 2.0, Excel 2007 Microsoft add in). AUCs were calculated using an NCA by the linear trapezoidal rule, and a uniform weight was performed as a first approach. Graphical concentration–time curves were produced after Log transformation. The ke was estimated from the terminal part of the log-concentration–time plot including at least three data points excluding the C_max_. 

### 2.7. Cell Lines

Gli36ΔEGFR-2 and U87-MG were used as GBM in vitro models. Gli36ΔEGFR [27,28], carrying the EGFRvIII mutation, was made resistant to TMZ after 1 month of in vitro exposure to 50 µM TMZ [29]. These cells were selected because the TMZ sensitivity was repeatedly tested in vitro and in vivo in orthotopic GBM models [30]. Both Gli36ΔEGFR-2 and U87-MG were maintained in Dulbecco’s Modified Eagle Medium (DMEM) High Glucose w/o sodium pyruvate (ECM0101L, Euroclone, Milan, Italy) supplemented with 10% fetal bovine serum (FBS, ECS0180L, Euroclone, Milan, Italy), 4 mM L-glutamine (ECB3000D, Euroclone, Milan, Italy), and 100 U ml^−1^ penicillin/streptomycin (P/S) (ECB3001B, Euroclone, Milan, Italy). Normal human astrocytes (NHAs, CC-2565, Lonza, Basel, Switzerland) were used as healthy astrocytes and maintained in AGM^TM^ Astrocyte Growth Medium BulletKit^TM^ (CC-3186, Lonza) as per the manufacturer’s protocol. Human cerebral microvascular endothelial cells (hCMECs), provided by Dr. S. Bourdoulous (Institut Cochin, Inserm, Paris, France), were used as a model of brain endothelial cells and cultured as reported in the literature [31]. Human umbilical vein endothelial cells (HUVECs, purchased from Lonza) were used as a model of peripheral endothelium and maintained in an Endothelial Cell Basal medium EGM^TM^ SingleQuots^TM^ Kit (CC-4133, Lonza) as per the manufacturer’s protocol. All cell lines were maintained at 37 °C with 5% CO_2_ and saturated humidity.

### 2.8. Cell Viability Assay and Targeting Efficacy of Liposomes

The effect of free or encapsulated Givinostat was assessed by the MTT assay. Cells were seeded in 96-well plates at a density of 2 × 10^4^ (Gli36ΔEGFR-2 and NHA) or 3 × 10^4^ (hCMEC/D3 and HUVECs) cells/well. Different doses of Givinostat, ranging from 0.1 to 20 μM, free or encapsulated in liposomes, were added to the culture medium for up to 72 h. Culture medium alone or added with DMSO or EtOH or unloaded liposomes (0.153 mM) were used as controls. At the designated times, the assay was performed as per the manufacturer’s protocol and absorbance was measured at 570 nm using a microplate reader (SPECTROstar Nano, BMG LABTECH, Ortenberg, Germany). Results are presented as the mean of three independent experiments ± SD. The IC_50_ mean value at 24 h was calculated basing on the relative viability values and concentrations using linear regression analysis provided by GraphPad Prism 8.

The targeting efficacy of LIP/m-GIV vs. LIP-GIV was evaluated by fluorescent techniques. Gli36∆EGFR-2 cells were seeded in a 96-well Cell Carrier Ultra plate (Perkin Elmer) at a density of 2.0 × 10^4^ cells/well. The actin cytoskeleton was labeled with CellMask™ Deep Red Actin Tracking Stain (1:1000) for 30 min. After washing with PBS, cells were incubated with Hoechst for 8 min as per the manufacturer’s protocol and then cells were washed again. Finally, cells were treated with fluorescent-labeled LIP or LIP/m (400 nM total lipids) for 15 min. Images were acquired using the Operetta CLS High Content Analysis System (Perkin Elmer, Waltham, MA, USA) equipped with 40x water objective and standard instrument filters as per the manufacturer’s protocol using the live imaging tool.

Quantitative analysis was performed by measuring the ratio between the fluorescence intensities in cell medium and cell lysates. Measurements were performed using a Spectrofluorometer FP-8500, Jasco, Tokyo, Japan.

### 2.9. Caspase-3 Activity by Immunofluorescence

An increase in cleaved Caspase-3 was determined by immunofluorescence microscopy and images were acquired using the Operetta CLS High Content Analysis System (Perkin Elmer) equipped with 40× water objective and standard instrument filters as per the manufacturer’s protocol. Gli36ΔEGFR-2 cells were seeded on a rat tail collagen I-coated 96-well Cell Carrier Ultra plate (Perkin Elmer) at a density of 1.5 × 10^4^ cells/well. Cells were treated with Givinostat for 48 h, washed with PBS, and fixed with 100 μl of 4% (*v/v*) formaldehyde for 15 min at RT. Then, cells were permeabilized with 0.5% Triton X-100 in PBS (*v/v*) for 5 min at RT and blocked with 3% bovine serum albumin (BSA) in PBS for 30 min. Cleaved Caspase-3 was stained using Alexa Fluor^®^ 488-conjugated anti-caspase-3 antibody (0.75 μg/mL in 3% BSA in TBS) (9669, ThermoFisher, Waltham, MA, USA) overnight at 4 °C. Actin cytoskeleton was stained with Phalloidin AlexaFluor^®^ 633 (1:100 in PBS) (Invitrogen, Waltham, MA, USA ) for 1 h at RT. Nuclei were stained with DAPI (1:1000 in PBS) (ThermoFisher) for 10 min at RT. Quantitative measurements of Caspase-3 accumulation after treatment were calculated as histograms’ intensity of the green channel images, fixing the threshold to >800 a.u.

### 2.10. Evaluation of HDACs Activity by Fluorescence Assay

Gli36ΔEGFR-2 cells were seeded in a 6-well plate at a density of 3 × 10^5^ cells/well, treated with Givinostat 0.5 μM, and lysed at different time points (10 and 30 min). Cell fractions (nucleus and cytoplasm) were extracted using an NE-PER^TM^ Nuclear and Cytoplasmatic Extraction Kit (cat. no. 78835, ThermoFisher) as per the manufacturer’s protocol. The reliability of cell fractions was assessed by Western blot, using GAPDH and Histone H3 primary antibodies (Figure S3). Then, the activity of HDACs was evaluated with an HDAC Activity Assay Kit (Fluorometric) (Ab156064, Abcam, Cambridge, UK) as per the manufacturer’s protocol. Data were obtained using a microplate reader (FLUOstar Omega, BMG LABTECH, Ortenberg, Germany) with continuous measuring every minute for 1 h at 380/460 nm.

### 2.11. Immunoblot Analysis

Cells were seeded in a 6-well plate at a density of 3 × 10^5^ cell/well and treated with Givinostat (0.25, 0.5 and 1 μM) for 48 h. Whole cell lysates were obtained by washing cells twice in cold PBS and harvesting in 60 μL of radioimmunoprecipitation assay (RIPA) buffer (cat. no. 89901, ThermoFisher) supplemented with 1% of protease and phosphatase inhibitor cocktail (cat. no. 78446, ThermoFisher). Whole cell lysates were quantified using a BCA Protein Assay Kit (cat. No. 23227, ThermoFisher) and separated by electrophoresis through precast gels (NuPAGE^TM^ 4–12% Bis-Tris, 1.0 mm, Mini Protein Gel 10 or 15-wells, cat. No. NP0321 and NP0323, ThermoFisher). 

Proteins were transferred to nitrocellulose membranes using iBlot^TM^ Transfer Stack (cat. No. IB301002, ThermoFisher) and membranes were blocked either in 5% milk or in 5% BSA in TBS with 0.1% Tween-20 (TBST) for 1 h. Membranes were then incubated overnight at 4 °C with the following primary antibodies: Acetyl-α-Tubulin (5335, 1:1000), α-Tubulin (2144, 1:1000), Histone H3 (9715, 1:1000) and β-Tubulin (2146, 1:1000) purchased from CST; β-Actin (MA5-15739, 1:5000), GAPDH (MA1-16757, 1:5000), VLDLR (MA5-24790, 1:1000), ABCA1 (PA1-16789, 1:500), LRP1 (MA1-27198, 1:500), and LDLR (PA5-22976, 1:1000) purchased from ThermoFisher. Membranes were incubated with secondary anti-rabbit antibody (A0545, 1:5000, Merck) or anti-mouse antibody (G21040, 1:20,000, Invitrogen) for 1 h at RT. Bands were detected using Immobilion ECL Ultra Western HRP Substrate (WBULS0100, Merck) under chemiluminescence using an Amersham Imager 600 (Cytiva, Marlborough MA, USA). Quantifications were made using ImageLab Software Version 6.1 (Bio-Rad, www.bio-rad.com).

### 2.12. 3D-Bioprinted GBM Models

The 3D-bioprinted GBM model was generated as already reported [32]. Briefly, a hybrid ink based on gelatin (GE-MF) and chitosan (CH-MF) was generated by Diels Alder crosslinking with maleimido-star-PEG (PEG-Star-MA). GE-MF (66 mg) and CH-MF (34 mg) were dissolved in 1.5 mL of PBS at 37 °C and vortexed until complete dissolution. PEG-Star-MA (5 mg) was dissolved in 0.5 mL of PBS at RT, added to the GE-CH hybrid solution, and mixed. The GE-CH solution was left for 30 min under UV-light for further sterilization and 2 h at 37 °C to obtain partial network formation of the hydrogel solution. U87-MG or Gli36ΔEGFR-2 cells (700 rpm centrifuge) (2 × 10^5^/mL) in complete medium were added to the GE-CH solution (5%, 2 mL) and transferred into a 5 mL bioprinter syringe. Each sample was bioprinted as a cylinder on 35-mm Petri TC dishes using a 22 G nozzle with a 0.41 mm diameter at 50 KPa. After printing, cells were maintained at 37 °C with 5% CO_2_. The culture media were refreshed every 2 days.

### 2.13. Drug Testing and Cell Viability in 3D-Bioprinted Models

Drug testing in 3D-bioprinted models was performed to test the effect of the following samples on Gli36ΔEGFR-2 and U87-MG viability: (1) empty liposomes as controls; (2) LIP-GIV; and (3) LIP/m-GIV. Cells were treated with 1 μM Givinostat at days 1 and 7 in MEM culture medium (2 mL per 35-mm dish) for 24 h. 

The cell viability in the 3D-bioprinted constructs after treatments was evaluated using a LIVE/DEAD^TM^ viability/cytotoxicity kit (Invitrogen), following the manufacturer’s instructions. In total, 1 mL LIVE/DEAD stock solution was added to each bioprinted construct. After 50 min of incubation at 37 °C, the stained bioprinted models were washed three times with PBS before image acquisition. Imaging analysis was performed with a confocal microscopy 10× or 20× Ph objective. Nuclei were stained using DAPI (1:1000 in PBS) (ThermoFisher) for 10 min at RT; living cells and dead cells were stained using calcein and EthD provided by the kit. Cell viability was calculated as ((number of green/red stained cells/number of total cells) × 100) using Fiji ImageJ Software [33].

Cell viability was also evaluated by Alamar assay to estimate the viability and/or mortality. In total, 200 μL of Alamar Blue solution (10% final volume) was added to each bioprinted sample and incubated for about 2 h. Absorbance was read at 570 nm at t0 (2 h of incubation), 24, and 48 h [34]. Results are presented as mean of five independent experiments ± SD.

### 2.14. Statistical Analysis

Statistical analysis was performed with GraphPad Prism 8, using the following tests: Two-way ANOVA, one-way ANOVA, unpaired t test, Sidak’s multiple comparisons test, and Tukey’s multiple comparisons test. Statistical significance was considered at *p* < 0.05.

## 3. Results and Discussion

### 3.1. Liposomes Improved the Pharmacokinetics Profile of Givinostat

**Figure 1:**
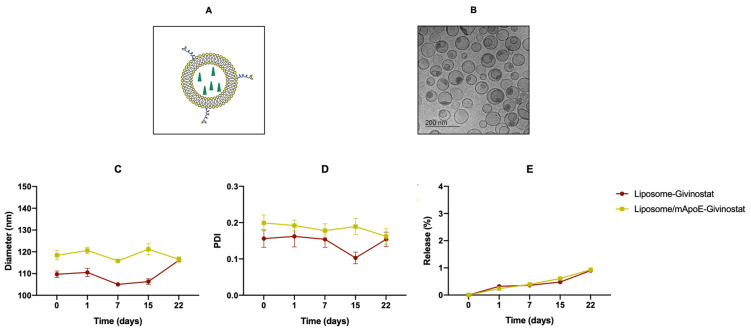
(**A**) Representative drawing showing a liposome loaded with Givinostat (green triangle) and functionalized with mApoE. (**B**) Cryo-EM image of drug-loaded vesicles with diameters ranging from 30 to 100 nm. Black dots on top and inside the vesicles indicate a high drug loading efficiency. (**C**–**E**) The diameter, polydispersity index, and drug release of LIP-GIV and LIP/m-GIV were measured around a period of three weeks. Data are presented as the mean of at least three independent experiments ± SD.

Liposomes composed of cholesterol/sphingomyelin/DSPE-PEG-mal, embedding Givinostat and surface functionalized with mApoE (Figure 1A), were prepared using the lipid film hydration method followed by extrusion, and were characterized by DLS. The results (Table 1, Figure S4) showed that liposomes had a uniform size distribution (PDI < 0.2), with a diameter <200 nm. The ζ-potential measurement showed that the net surface charge of liposomes was negative. This suggests that the dispersions are stable and not prone to aggregation. A slight increase in the size (+8%) was detected after surface functionalization with mApoE. These parameters are indicative of homogenous samples with a stable profile. The EE% was 84 ± 11% and 92 ± 4% while the D/L (μg/μg) was 0.27 ± 0.15 and 0.52 ± 0.35 for LIP-GIV and LIP/m-GIV, respectively (n = 8).

A representative cryo-EM image of the liposomes is shown in Figure 1B. The image reveals spherical, unilamellar vesicles homogeneously distributed in vitreous ice, with diameters ranging from 30 to 100 nm. Black dots on the top and inside the vesicles indicate a high loading efficiency of the drug. 

The stability of liposomes was determined by following the size, PDI, ζ-potential, and drug release over three weeks. Results showed that the size (Figure 1C) and PDI (Figure 1D) of the liposomes as measured by DLS did not undergo significant changes. The ζ-potential and drug release remained <−20 mV and <0.9% (Figure 1E), respectively, for both formulations.

We next investigated the effect of the liposome preparations on endothelial cell viability using the MTT assay on hCMEC/D3 and HUVEC cell lines as models of brain and peripheral endothelium, respectively (Figure S5). The cell viability after treatment with LIP-GIV or LIP/m-GIV was >50% for all the conditions tested, similar to the free drug. Moreover, 50% mortality was reached only at the highest dose (20 μM) of liposomes-Givinostat on hCMEC/D3 cells. Considering that, in other studies, the pharmacological effect of Givinostat on GBM cells was obtained at doses ranging between 0.25 and 5 µM [12], liposome preparations containing that concentration of drug can be considered harmless for endothelia. This is in accordance with the results obtained by Milan M. et al. about the potential protective effect of Givinostat on blood vessels from apoptosis [35].

**Figure 2:**
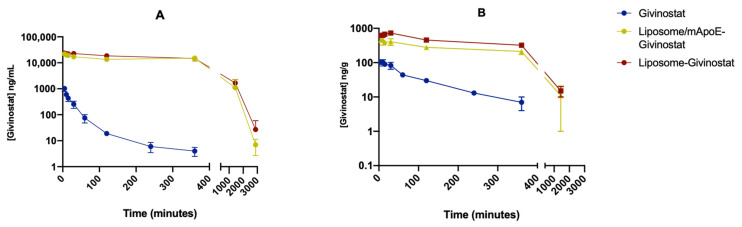
Concentration of Givinostat detected in plasma and brain after i.v. administration of free Givinostat, LIP-GIV, or LIP/m-GIV. (**A**) Plasma concentration of Givinostat from 5 min to 48 h post-injection. (**B**) Brain concentration of Givinostat from 5 min to 48 h post-injection. Data were obtained through the LC-MS/MS method.

The pharmacokinetic parameters of free Givinostat or of the compound embedded in liposomes were measured in healthy animals. The mean plasma concentrations of Givinostat, after a single i.v. administration, are shown in Figure 2A (all concentrations are available in Table S2). LIP-GIV and LIP/m-GIV extended the half-life of free Givinostat (t_1/2_ = 1.7 h) to 5.1 and 3.8 h, respectively. At 6 h, the concentrations of LIP preparations in plasma were approximately 15,000 ng/mL. In contrast, free Givinostat was rapidly removed from the circulation and could not be detected 24 h after i.v. administration. Liposomal formulations of Givinostat remained in the blood circulation up to 48 h post-injection and showed delayed plasma clearance, in comparison to the free drug. 

A strong increase in plasma exposure (AUC_0-t_) (AUC_Givinostat_ = 478 ng/mL h; AUC_LIP-GIV_ = 276,825 ng/mL h; AUC_LIP/m-GIV_ = 249,919 ng/mL h) was detected for LIP formulations, indicating lower plasma clearance (CL) was exhibited by LIP formulations compared to free Givinostat (CL_Givinostat_ = 15,417 mL/kg*h, CL_LIP-GIV_ = 27 mL/kg*h, and CL_LIP/m-GIV_ = 30 mL/kg*h). In addition, an increase in the mean residence time (MRT_0-t_) was observed with LIP (MRT_Givinostat_ = 0.6 h; MRT_LIP-GIV_ = 7 h; MRT_LIP/m-GIV_ = 6.6 h). The volume of distribution of free Givinostat is 10.8 L/kg, largely exceeding the total body water, while the volume of distribution of the liposome formulations is significantly lower (~0.2 L/kg). The increase observed for the AUC and MRT values could be due to the liposomal composition. Specifically, PEGylation is a feature that may confer stealth properties to liposomes. These results agree with other published data showing the ability of liposomes to enhance drug stability and its persistence in plasma by limiting the adsorption of blood components onto their surface [36]. Taken together, these data are similar to those previously described for liposomal formulations of Vincristine [37].

The amount of Givinostat measured in the brain is shown in Figure 2B (all concentrations are available in Table S3), suggesting that the encapsulation of Givinostat in liposomes improved drug delivery to the brain. Both liposomal formulations led to a brain concentration of Givinostat of approximately 270 ng/g, 6 h after dosing. In contrast, the administration of Givinostat in the PEG/DMSO vehicle led to a brain tissue concentration of only 7 ng/g at the same time point, likely due to its high clearance rate from the systemic circulation. Then, 24 h after administration, Givinostat levels were below detection limits in the PEG/DMSO vehicle group while 15 ng/g of Givinostat was found in the brain of animals that received the liposomal formulations. The liposomal formulations showed a strong increase in the total exposure (AUC) of Givinostat that reached the brain, being much higher than the free drug (ratio from 20 to 30 times). On the other hand, the AUC_brain_/AUC_plasma_ ratio for free Givinostat was 0.372, higher than liposomal formulations ratios (0.020 for LIP-GIV and 0.014 for LIP/m-GIV). As Givinostat is a hydrophobic small molecule (~400 Da), it is able to cross the BBB by simple diffusion, unlike liposome formulations, which cross the BBB through endo/transcytosis [38]. In accordance with the results obtained for Vincristine entrapped in liposomes [37], we showed that the incorporation of Givinostat in liposomes should improve the therapeutic index by increasing the duration of drug exposure to the target tissue. 

In vivo, Givinostat gives rise to two main metabolites, deriving from the biotransformation of the hydroxamic acid mediated by different enzymes: the hydroxamate moiety may be hydrolyzed into a carboxylic acid (ITF2375) or reduced into an amide (ITF2374) [39]. The metabolites inhibit HDACs at concentrations from three to five orders of magnitude higher than Givinostat’s and, in preclinical models, they do not contribute to its efficacy [40]. ITF2375 was the most abundant metabolite in plasma, in comparison to the ITF2374 compound (Figure S6, panels A,B and Table S2), as reported for free Givinostat [40]. In contrast, ITF2374 levels in the brain were slightly higher (Figure S6, panels C,D and Table S3). Accordingly, the ratio between ITF2374 and ITF2375 after 1 h of Givinostat administration was 0.18 and 5.6 in the plasma and brain, respectively. This difference could be due to the different enzymatic expression in the brain and peripheral tissues, or to the diverse grade of brain penetration of the two metabolites, but this hypothesis needs to be confirmed. The difference detected at the starting point between free and liposome-encapsulated Givinostat was not observed for the metabolites. This could be explained considering that only the free Givinostat fraction is metabolizable as it is accessible to enzymes, unlike when the drug is incapsulated in liposomes.

No significant difference in the pharmacokinetics and brain penetration between LIP-GIV and LIP/m-GIV was found. Indeed, both preparations were able to increase C_max_ in the brain of about 6-fold over free Givinostat (~700 and ~500 vs. ~100 ng/g, respectively). However, it is important to note that in previous work, we demonstrated an increase in mApoE receptors on both BBB and GSCs after GBM mice irradiation [41]. This information should play in favor of using LIP/m-GIV to increase the delivery of Givinostat to cancer cells in GBM in vivo models. Nevertheless, both liposomal formulations are valid tools to deliver Givinostat to the brain and to increase its persistence in plasma. However, further studies on tumor-bearing animal models are needed because the presence of a tumor can impact on the circulation time and biodistribution of liposomes, as shown for polymersomes [42].

### 3.2. Givinostat Embedded in Liposomes Maintained Its Anti-Tumor Activity in 2D and 3D In Vitro Models

Givinostat has shown anti-cancer activity on various tumor cell lines [7,43,44], but the literature concerning GBM is scarce. We evaluated the cytotoxic activity of Givinostat on the Gli36ΔEGFR-2 cell line, expressing the EGFRvIII variant that is present on up to 54% of cells isolated from GBM patients (mean = 28–30%); it is one of the most frequent genetic aberrations associated with GBM (Figure S7) [45,46,47]. Thus, this cell line represents an appropriate in vitro model to study the disease. In parallel, the effect of Givinostat was also tested on NHA cells, used as healthy controls. 

**Figure 3:**
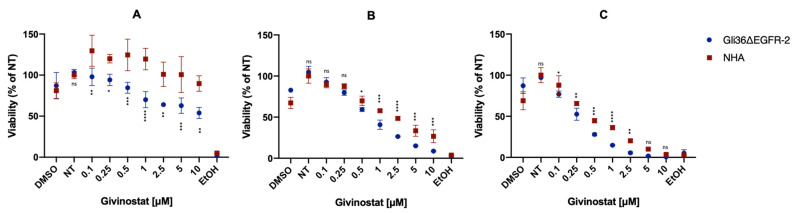
Evaluation of the cytotoxicity on Gli36ΔEGFR-2 and NHA cell lines treated with free Givinostat for 24 (**A**), 48 (**B**), or 72 h (**C**). Control DMSO was given in equivalent microliters than the highest dose of the inhibitor. Half an hour before the assay, three wells were pre-treated with 100% EtOH to provide a near 100% mortality as a control. NT were established as controls at 100% viability. Each graph is the result of three independent experiments. Ns, not significant; *, *p* < 0.05; **, *p* < 0.01; ***, *p* < 0.001; ****, *p* < 0.0001, two-way ANOVA, Sidak’s multiple comparisons test.

Gli36ΔEGFR-2 and NHA cell lines were first treated with the free drug and the cell viability was determined using the MTT assay (Figure 3). The toxicity of Givinostat was both dose- and time-dependent, and the drug seemed to exhibit a natural selectivity for cancer cells versus healthy cells, which was maintained up to the dose of 2.5 μM and until 72 h of treatment, even if the differences were slight. However, the NHA cell viability was equal to or higher than 100% only after 24 h of treatment with Givinostat doses ranging between 0.1 and 10 μM. It is possible to speculate that this selectivity is also maintained in vivo. After a single i.v. injection, Givinostat embedded in liposomes was still present in the brain after 24 h while it was undetectable after 48 h. If the drug selectivity in vivo was confirmed in future studies, Givinostat should be able to induce GBM cells’ mortality without affecting healthy cells after the first administration. The selective cytotoxicity of HDACis on transformed cells has been described by others and several mechanisms have been invoked, such as differences in cell cycle checkpoints [48] or higher concentrations of reactive oxygen species (ROS) in cancer cells treated with HDACis. In fact, Bolden et al. [49] demonstrated that cancer cells accumulate ROS more easily compared to healthy cells after treatment with the HDACi Vorinostat. Moreover, previous reports [50,51] have noted that the different epigenetic regulations and gene expressions in cancer cells might be correlated to the cancer-selective cytotoxicity of HDACis. The mean calculated IC_50_ in Gli36ΔEGFR-2 cells after 24 h of treatment was 0.75 μM. Considering that the IC_50_ for TMZ on the U87-MG and T98G cell lines is in the range of 100–500 μM [52], we speculate that Givinostat could be a promising adjuvant or alternative chemotherapeutic drug, especially in TMZ-resistant cells. 

**Figure 4:**
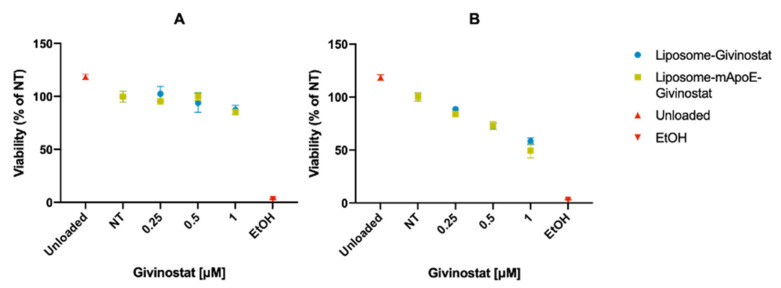
Evaluation of the cytotoxicity on the Gli36ΔEGFR-2 cell line treated with LIP-GIV or LIP/m-GIV for 24 (**A**) or 48 h (**B**). The “unloaded” sample represents the unfunctionalized liposome without any drug loaded (lipid concentration ~8 μM, same lipid concentration as the 1 μM dose). Half an hour before the assay, three wells were pre-treated with 100% EtOH to provide a near 100% mortality as a control. NT were established as controls at 100% viability. Each graph is the result of three independent experiments ± SD.

To evaluate if Givinostat retains its cytotoxic activity after incorporation in liposomes, we performed an MTT assay on Gli36ΔEGFR-2 cells after treatment with LIP-GIV and LIP/m-GIV. Three low doses were chosen, considering the improved blood half-life and brain uptake of the drug incorporated in liposomes (see above). The results (Figure 4) showed that the encapsulation of the drug in liposomes did not affect its anti-tumor action, preserving the dose- and time-dependent cytotoxic activity. However, it is noteworthy that only LIP-GIV reduced free Givinostat activity at the highest dose and time tested (*p* = 0.0042). In addition, it is important to point out that considering the increased plasma half-life of liposome formulations in comparison to the free drug and the low toxicity on endothelial cells, an enhancement of the anti-tumor efficacy by increasing the tumor drug deposition and a reduction in side effects might be expected [53].

**Figure 5:**
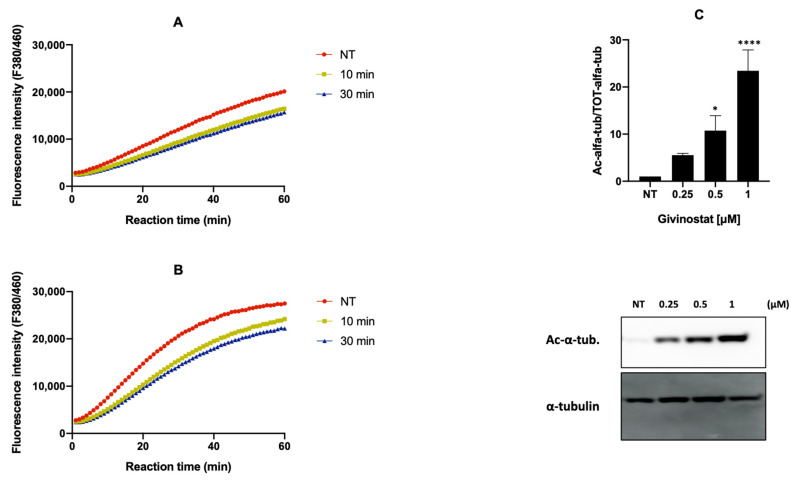
Percentage of HDACs activity in the Gli36ΔEGFR-2 cell line treated with LIP/m-GIV 0.5 μM in the cytoplasm (**A**) and nucleus (**B**). After each selected time, cells were lysed, and cell fractions were extracted. HDAC activity was established through an HDAC activity assay based on ligand fluorescence. (**C**) The effect of LIP/m-GIV on α-tubulin acetylation. WB showing the protein levels of acetylated-α-tubulin (Ac-α-tub.) and total α-tubulin in Gli36ΔEGFR-2 cells without treatment (NT) or treatment with LIP/m-GIV in various concentrations. The graph shows the quantifications of Ac-α-tub. in three independent experiments. Total α-tubulin was used as a normalization protein. *, *p* < 0.05; ****, *p* < 0.0001, one-way ANOVA, Tukey’s multiple comparisons test.

We also investigated if the liposomal formulations maintained the HDAC inhibitory effect of Givinostat in Gli36ΔEGFR-2 cells. We demonstrated that the activity of both cytosolic and nuclear HDACs was reduced after only 10 min of treatment with 0.5 μM LIP/m-GIV, reducing the activity by 25% after 30 min of treatment (Figure 5, panels A,B). This indicates that liposomal formulations are internalized inside cells within a few minutes, facilitating rapid action by Givinostat on HDACs. As a reminder, HDACs1-3 are exclusively nuclear while the others are either mostly cytoplasmic (HDAC6 and 10) or shuttle between the cytoplasm and nucleus [54,55]. Accordingly, we detected a strong dose-dependent increase in α-tubulin acetylation in treated Gli36ΔEGFR-2 (Figure 5C). Of note, cytosolic HDAC6 is a microtubule-associated protein whose task is to deacetylate non-histonic proteins such as α-tubulin [56], thus explaining the increase in its acetylation induced by the treatment with LIP/m-GIV. 

Traditionally, anti-cancer drugs have been evaluated in conventional 2D cell culture systems that poorly mimic the complexity and heterogeneity of in vivo tumors, which usually grow in 3D [57]. The limitations of 2D in vitro models include the absence of the GBM microenvironment (especially ECM components), very different culture conditions reported in the literature, and unphysiological oxygen levels, beyond the loss of the intrinsic in vivo heterogeneity of the tumor [58,59]. Although in vivo studies remain a fundamental step in cancer research, animals often do not represent a realistic model of GBM when human xenograft or orthotopic transplants are used. In fact, they are different than the original niche, do not show an infiltrative nature as human GBM does, and immunomodulatory therapies cannot be tested [60,61]. Moreover, mice do not exhibit endothelial proliferation and, more importantly, when xenograft transplantations are performed through subcutaneous injection, the local microenvironment is very different from the brain microenvironment, resulting in a lack of tumor growth [62]. Given these premises, several 3D in vitro models have been developed as a surrogate or a complementary approach to classic ones for evaluating drug efficacy [63]. They represent a valid compromise between the lack of complexity and heterogeneity of 2D in vitro models and the claims emerging from in vivo GBM models [64]. Although some limitations still need to be overcome [65], tumor cells (GBM cells included) grown in a 3D scaffold better recapitulate the features of patient-derived cells, in comparison to 2D culture conditions [66,67].

**Figure 6:**
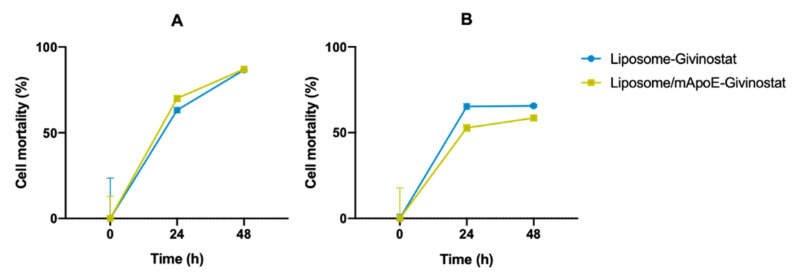
Alamar blue assay on bioprinted U87-MG (**A**) or Gli36ΔEGFR-2 (**B**) gelatin-chitosan hydrogels after liposome administration. Alamar blue solution (10% final volume) was added to each sample and incubated for approximately 2 h. Absorbance was read at 570 nm at the selected timepoints. Empty liposomes were employed as a positive control. Results are presented as five independent experiments ± SD. All data were normalized with the positive control absorbance values obtained.

Accordingly, we tested the ability of LIP-GIV and LIP/m-GIV to affect the viability of 3D-bioprinted constructs generated using U87-MG and Gli36ΔEGFR-2 cells. Seven days after printing, 3D-bioprinted cells were treated for different times with liposomes and inhibition of cell growth was measured using the Alamar and LIVE/DEAD assays. Results showed that both LIP-GIV and LIP/m-GIV were able to affect the viability of both U87-MG and Gli36ΔEGFR-2 cells (≥50% mortality) after 24 h of treatment in a 3D-printed model, as detected by the Alamar assay (Figure 6).

**Figure 7:**
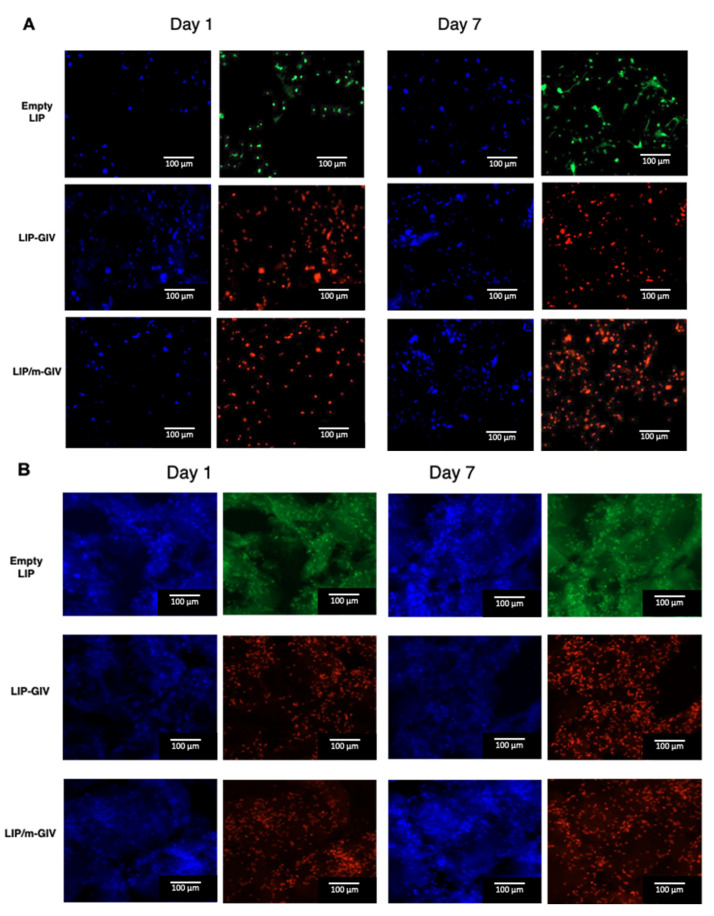
LIVE/DEAD microscopy images on day 1 and 7 after printing. U87-MG (**A**) or Gli36ΔEGFR-2 (**B**) cell lines were bioprinted with gelatin-chitosan hydrogel and cultured for 1 and 7 days. Then, empty liposomes, LIP-GIV, or LIP/m-GIV were administered for 24 h. Blue, DAPI (nuclei); red, EthD (dead cells); green, calcein (living cells). Cell viability was calculated as ((number of green-red stained cells/number of total cells) × 100) using Fiji ImageJ software.

These data were also confirmed by the images obtained following the LIVE/DEAD assay (Figure 7), where > 50% cell mortality was detected after 24 h of treatment for both liposome formulations tested. Quantifications (Table 2) showed only mild and non-significant differences between the two cell lines used. This could be due to the different profile of liposome endocytosis. However, these results confirm those obtained in 2D models and demonstrate that LIP-GIV and LIP/m-GIV are also able to target GBM cells in a model with a complex ECM-like network and a 3D structural organization. 

**Figure 8:**
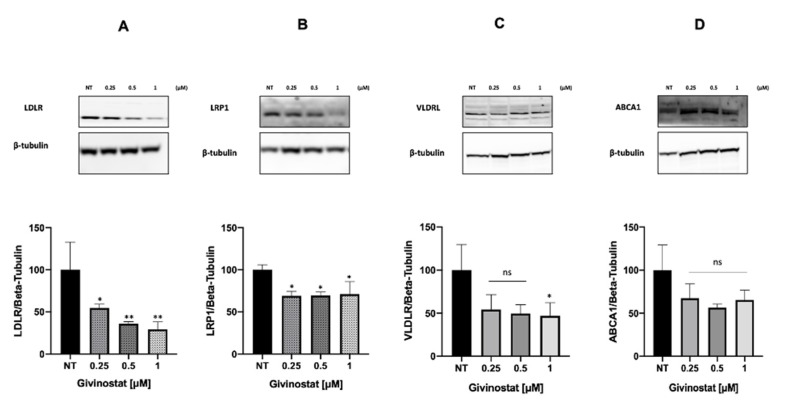
WB representing the effect of LIP-GIV on the expression of LDLR (**A**), LRP1 (**B**), VLDLR (**C**), and ABCA1 (**D**) receptors in Gli36ΔEGFR-2 after 24 h, with quantitative graphs. Each graph is the result of three independent experiments. β-tubulin was used as a normalization protein. Ns, not significant; *, *p* < 0.05; **, *p* < 0.01, one-way ANOVA, Tukey’s multiple comparisons test.

The functionalization of liposomes with mApoE was originally performed to promote their ability to reach the brain through the BBB in pathological conditions after mouse irradiation, as already shown in animal models [41,68,69]. In addition, this functionalization could also be exploited to increase the target selectivity towards tumor cells, because it has been shown that GBM cell lines overexpress low-density lipoprotein receptor (LDLR), very-low-density lipoprotein receptor (VLDLR), and low-density lipoprotein receptor-related protein 1 (LRP1), to which mApoE binds [70,71]. Indeed, increased cellular uptake of mApoE-liposomes was also detected for Gli36∆EGFR-2 (Figure S8). Nevertheless, our results showed that there is no difference in the cell viability between LIP-GIV and LIP/m-GIV (Figure 4). For this reason, we investigated the levels of LDLR, VLDLR, and LRP1 in Gli36ΔEGFR-2 cells. Interestingly, the results demonstrated that all three receptors were significantly reduced after treatment with Givinostat (Figure 8, panels A–C). These results can explain the comparable effect between functionalized and non-functionalized liposomes on Gli36ΔEGFR-2 viability. Moreover, to the best of our knowledge, the effect of Givinostat on these three receptors has never been shown and these findings increase the understanding of its mechanism of action. This additional feature may enhance the anti-tumor activity of Givinostat because the reduction in LDLR, VLDLR, and LRP1 levels can limit GBM survival by decreasing the uptake of lipoproteins, thus altering cell lipid metabolism. Their overexpression seems to be related to cancer progression; in fact, GBM cells are incapable of de novo cholesterol synthesis and their survival depends on cholesterol uptake by LDLRs [72]. Moreover, LRP1 expression has been linked to GBM cell migration and tumor invasion because it induces the expression of metalloproteases 2 and 9 via an ERK-dependent signaling pathway [73].

In addition, we investigated the expression of ATP-binding cassette protein A1 (ABCA1), which is involved in cholesterol efflux from astrocytes [71,74]. As shown in Figure 8D, there is a non-significant trend towards a decrease in ABCA1 expression after treatment with Givinostat. This might be expected as a compensatory mechanism due to the parallel decrease in LDLR, VLDLR, and LRP1 protein expression. In other words, it is reasonable to assume that the decreased cholesterol uptake may stimulate GBM cells to mildly limit their efflux and preserve cholesterol storage. However, it is important to highlight that the ability of Givinostat to reduce ABCA1 levels could also be a potential strategy for the treatment of TMZ-resistant GBM because it has been shown that TMZ efflux is controlled by ABCA1 activity [75]. Therefore, co-administration of Givinostat could potentiate the TMZ efficacy in TMZ-resistant GBM cells. These assumptions need further investigation. 

Another point to consider is that the reduction in LDLR expression induces substantial apoptosis in U87EGFRvIII cells, as reported by Villa G.R. et al. [76]. To investigate whether this process also occurs in Gli36ΔEGFR-2 cells after Givinostat treatment, the expression of cleaved Caspase-3 was evaluated by immunofluorescence. As shown in Figure S9, the fluorescence associated with cleaved Caspase-3 was detected after 48 h of treatment with Givinostat in a dose-dependent manner. Similar results at the same timepoints have been obtained on human lymphoblastic leukemia [44], supporting the hypothesis that Givinostat acts as an apoptosis-inducing drug. However, in other published data on human sarcoma [77], Givinostat induced apoptosis after 72 h of treatment and exhibited a tumor-selective pro-apoptotic activity that was prolonged over time. This issue deserves further investigations.

## 4. Conclusions

GBM is the most common malignant and lethal primary brain tumor. Herein, using 2D and 3D in vitro models, we showed that the pan-HDAC inhibitor Givinostat embedded in liposomes counteracts GBM cell growth by inducing: (1) a dose- and time-dependent reduction in cell viability; (2) a reduction in LDLR, LRP1, and VLDLR protein receptors; (3) a mild reduction in ABCA1 levels; and (4) an increase in cleaved Caspase-3. In addition, the incorporation of Givinostat in liposomes increased the drug half-life in the bloodstream and the amount of drug entering the brain in healthy animal models for both preparations tested. Thus, LIP-GIV and LIP/m-GIV, by acting as a cytotoxic drug that is able to cross the BBB, could be considered as a potential approach against GBM. However, additional pre-clinical studies need to be performed to make this liposomal product applicable in this field.

## Figures and Tables

**Table 1:** Physico-chemical characterization of LIP-GIV and LIP/m-GIV formulations at T0.

Liposomal Formulation	Diameter (nm) ± SD	PDI ± SD	ζ-Potential ± SD
LIP-GIV	109.7 ± 1.5	0.156 ± 0.024	−22.13 ± 1.51
LIP/m-GIV	118.4 ± 2.1	0.199 ± 0.022	−25.84 ± 1.87

**Table 2:** Cell mortality (% of control ± SD) in 3D-bioprinted models after treatment with LIP-GIV or LIP/m-GIV.

Liposomal Formulation	Day 1	Day 7
LIP-GIV (Gli36∆EGFR-2)	75 ± 13	78 ± 12
LIP/m-GIV (Gli36∆EGFR-2) LIP-GIV (U87-MG) LIP/m-GIV (U87-MG)	66 ± 22 81 ± 11 89 ± 24	77 ± 16 76 ± 3 91 ± 3

## Data Availability

The data presented in this study are available on request from the corresponding authors.

## Supplementary Materials

The following supporting information can be downloaded at: https://www.mdpi.com/article/10.3390/cancers14122978/s1, Table S1: MRM transitions and MS parameters of Givinostat and its metabolites; Figure S1: Chromatograms of Givinostat, ITF2374, and ITF2375 MRM transition; Figure S2: Calibration curves for Givinostat, ITF2374, and ITF2375 in plasma and brain homogenate; Figure S3: Reliability of cell fractions from Gli36ΔEGFR-2; Figure S4: Representative autocorrelation functions and CONTIN analysis of the particle size distribution for LIP-GIV and LIP/m-GIV; Figure S5: Evaluation of the cytotoxicity on hCMEC (A) or HUVEC (B) cell lines treated with LIP-GIV or LIP/m-GIV for 24 h; Table S2: Plasma levels of Givinostat and its metabolites; Table S3: Brain levels of Givinostat and its metabolites; Figure S6: Distribution of Givinostat metabolites in the brain and plasma after i.v. administration of free Givinostat, LIP-GIV, or LIP/m-GIV; Figure S7: Expression of EGFR in A549 and Gli36ΔEGFR-2 cell lines; Figure S8: Targeting efficacy of fluorescent-labeled liposomes on Gli36∆EGFR-2; Figure S9: Increase in cleaved Caspase-3 in Gli36ΔEGFR-2 cells after treatment with Givinostat.
