# Supplementary material for: Givinostat-Liposomes: Anti-Tumor Effect on 2D and 3D Glioblastoma Models and Pharmacokinetics
Source: Cancers (Basel). 2022 Jun 16;14(12):2978. doi: 10.3390/cancers14122978 (PMC9220922; doi:10.3390/cancers14122978)

Original WB Figures of Figure 5C

Acetyl-α-tubulin

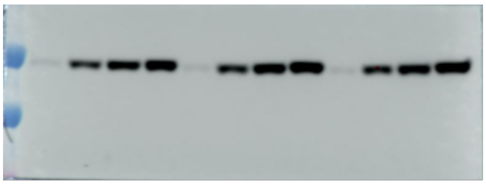

Total-α-tubulin

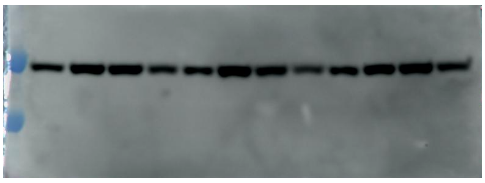

Quantifications

	Ac-alfa-tub	TOT-alfa-tub
NT	847840	13312863
0.25	8713920	25404515
0.5	14209420	23509080
1	18940016	13501485

	Ac-alfa-tub	TOT-alfa-tub
NT	1057336	14884043
0.25	10212104	27449070
0.5	18449816	18108775
1	24669315	12227842

	Ac-alfa-tub	TOT-alfa-tub
NT	1042775	14572827
0.25	9860256	23107788
0.5	16332609	27299654
1	27537246	19386720

Original WB Figures of Figure 8

LDLR

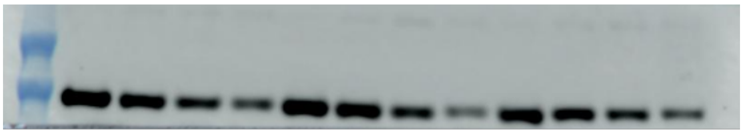

LRP1

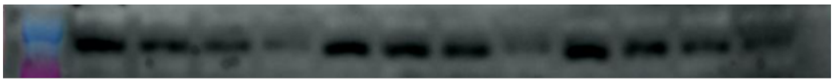

β-tubulin

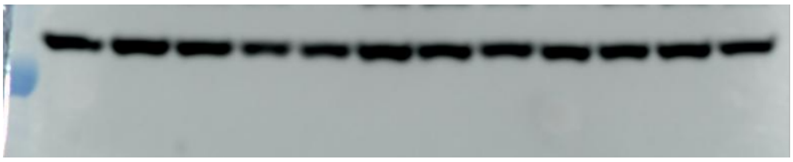

VLDLR

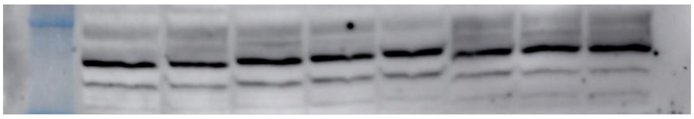

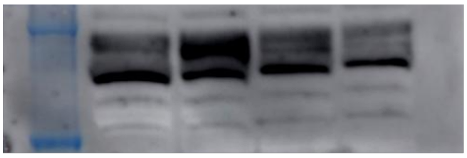

ABCA1

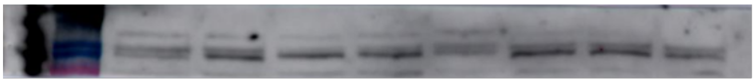

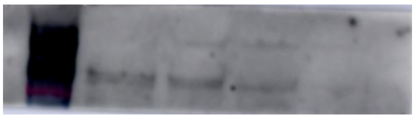

β-tubulin

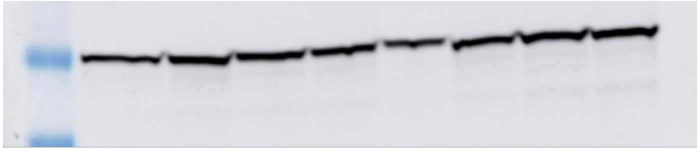

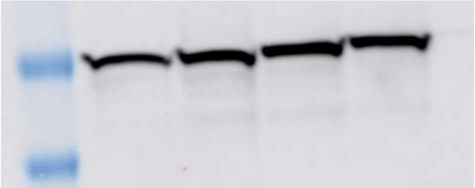

Quantifications

	LDLR	LRP1	B-Tub
NT	24262212	10753200	21058912
0.25	13587345	9265150	25018896
0.5	7809974	8116000	21609000
1	5793695	9360350	14766024

	LDLR	LRP1	B-Tub
NT	19378390	8572050	15987664
0.25	14398245	9762500	24451224
0.5	7988319	7486050	20940192
1	4227704	8369700	18675552

	LDLR	LRP1	B-Tub
NT	12951981	10705900	20896400
0.25	10118548	6885550	20237448
0.5	7172808	8313950	21789712
1	4754047	11087100	18462360

ABCA1			
NT	29793192	22385369	22968435
0.25	19140618	20621133	27197450
0.5	21350406	19250616	24680040
1	21471425	24723068	22406172

B-TUB			
NT	20893825	28567047	20955564
0.25	29048382	33392080	28578560
0.5	35911170	32225298	36646371
1	30153510	29042460	37348494

VLDLR			
NT	42869640	31438596	36697662
0.25	27803430	19142000	32240354
0.5	27731904	32030790	24241040
1	16026660	29843528	27738744

Original WB Figures of Figure S3

GAPDH

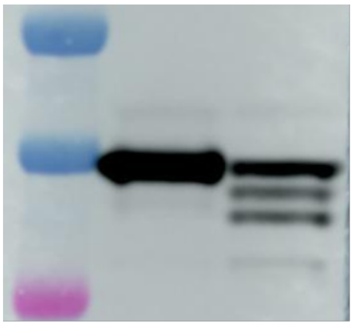

Histone H3

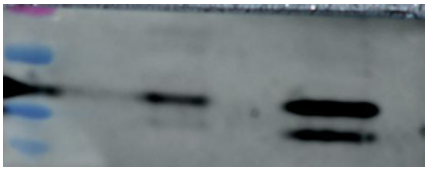

Original WB Figures of Figure S7

EGFR

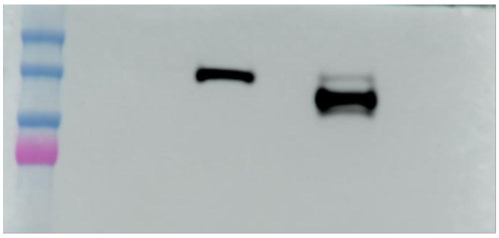

β-tubulin

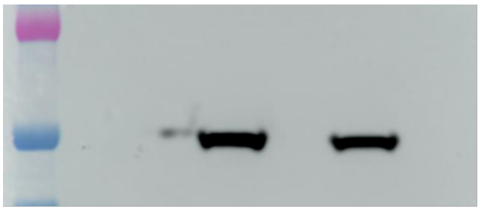

Supplement: Supplementary file 1 [file cancers-14-02978-s001.zip › cancers-1697072 _original_WB.pdf]
