# Supplementary material for: Givinostat-Liposomes: Anti-Tumor Effect on 2D and 3D Glioblastoma Models and Pharmacokinetics
Source: Cancers (Basel). 2022 Jun 16;14(12):2978. doi: 10.3390/cancers14122978 (PMC9220922; doi:10.3390/cancers14122978)
Supplement: Supplementary file 1 [file cancers-14-02978-s001.zip › cancers-1697072_supplementary_file.pdf]

SUPPLEMENTARY IMAGES

Table S1. MRM transitions for qualification and quantification and MS parameters of Givinostat and its metabolites

Compound	Parent Ion (Q1)	Product Ion (Q3)	Time	DP	EP	CE	CXP
ITF2357_1*	422.2	186.3	150	53	7.2	35	1
ITF2357_2	422.2	305.1	150	60	2.1	32.2	2
ITF2374_1*	406.2	170.2	150	47	4.2	41	1.4
ITF2374_3	406.2	333.3	150	47	5	28	4
ITF2375_1*	407.3	334.3	150	50	3.3	27	3
ITF2375_2	407.3	171.3	150	45	4.7	39	3.2
ITF2400_1*	436.2	186.4	150	59	3.2	32	1.4

T 550°C, Gas 1 50, Gas 2 50, CUR 25, CAD 2, IS 5500

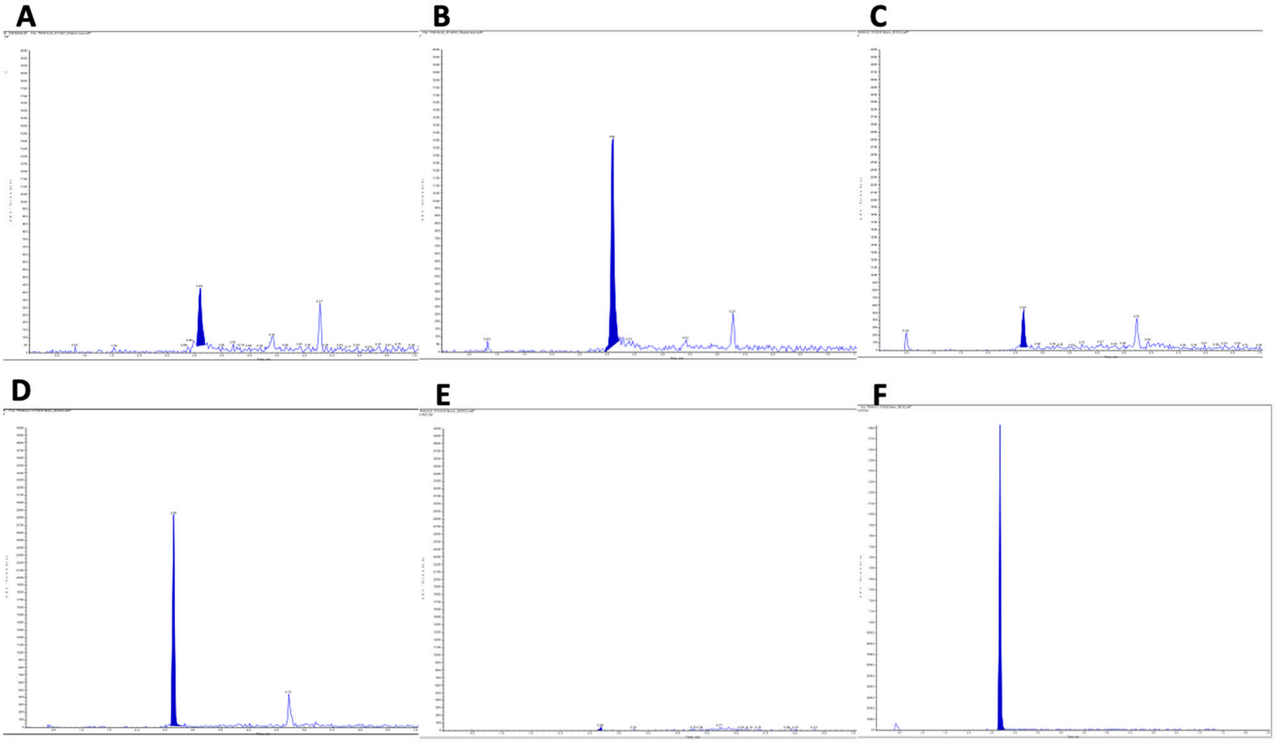

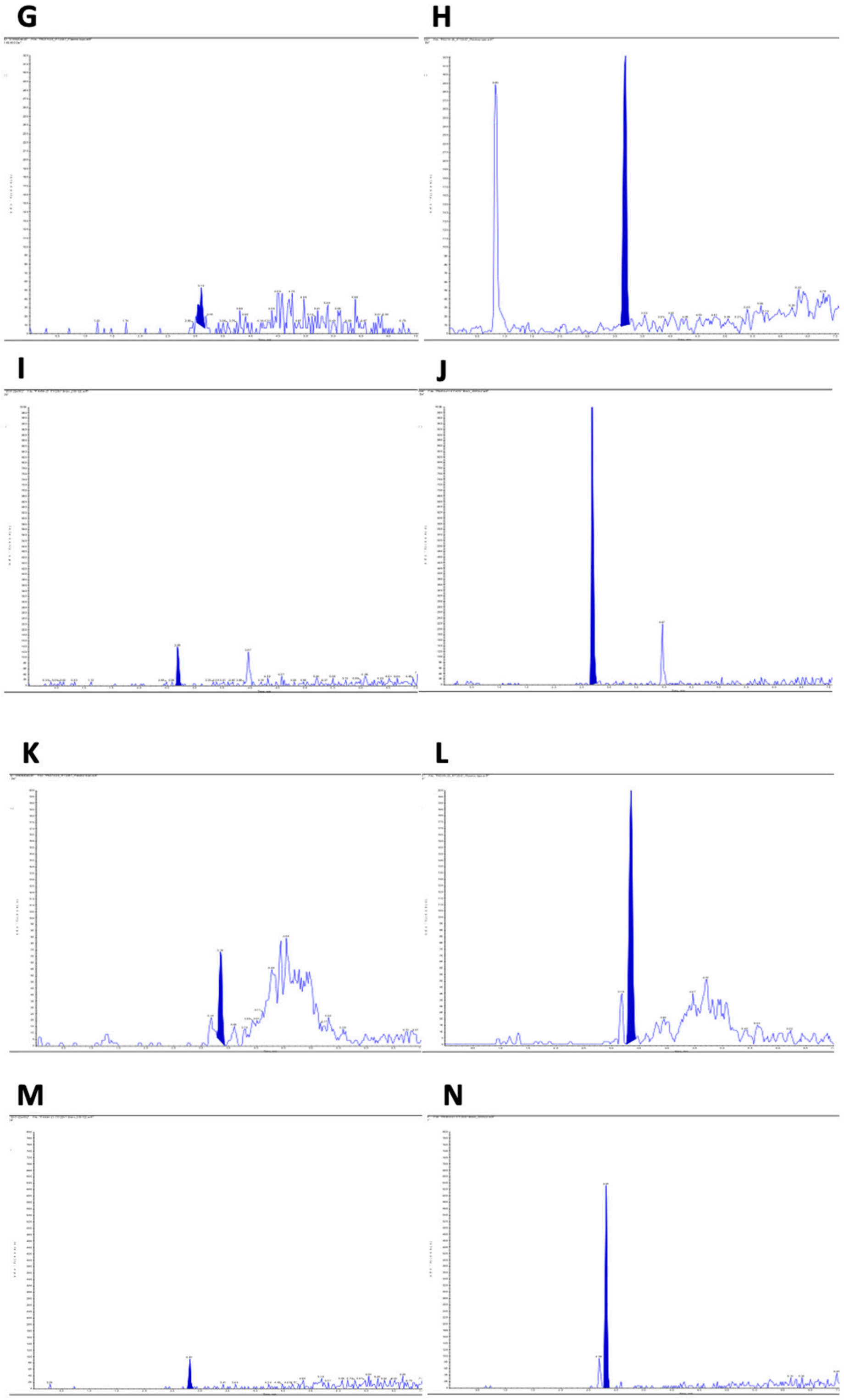

Figure S1. **Panels A-F**: representative chromatograms of Givinostat MRM transition 422.2/186.3 amu. **(A)** Blank plasma; **(B)** LLOQ 2.5 ng/mL in plasma; **(C)** Blank brain homogenate; **(D)** LLOQ in brain homogenate 0.5 ng/mL; **(E)** Internal standard ITF2400 MRM transition 436.2/186.4 amu; **(F)** Representative chromatogram in plasma. **Panels G-J**: representative chromatograms of ITF2374 MRM transition 406.2/170.2 amu. **(G)** Blank plasma; **(H)** LLOQ in plasma; **(I)** Blank brain homogenate; **(J)** LLOQ in brain homogenate. **Panels K-N**: representative chromatograms of ITF2375 MRM transition 407.3/334.3. **(K)** Blank plasma; **(L)** LLOQ in plasma; **(M)** Blank brain homogenate; **(N)** LLOQ in brain homogenate.

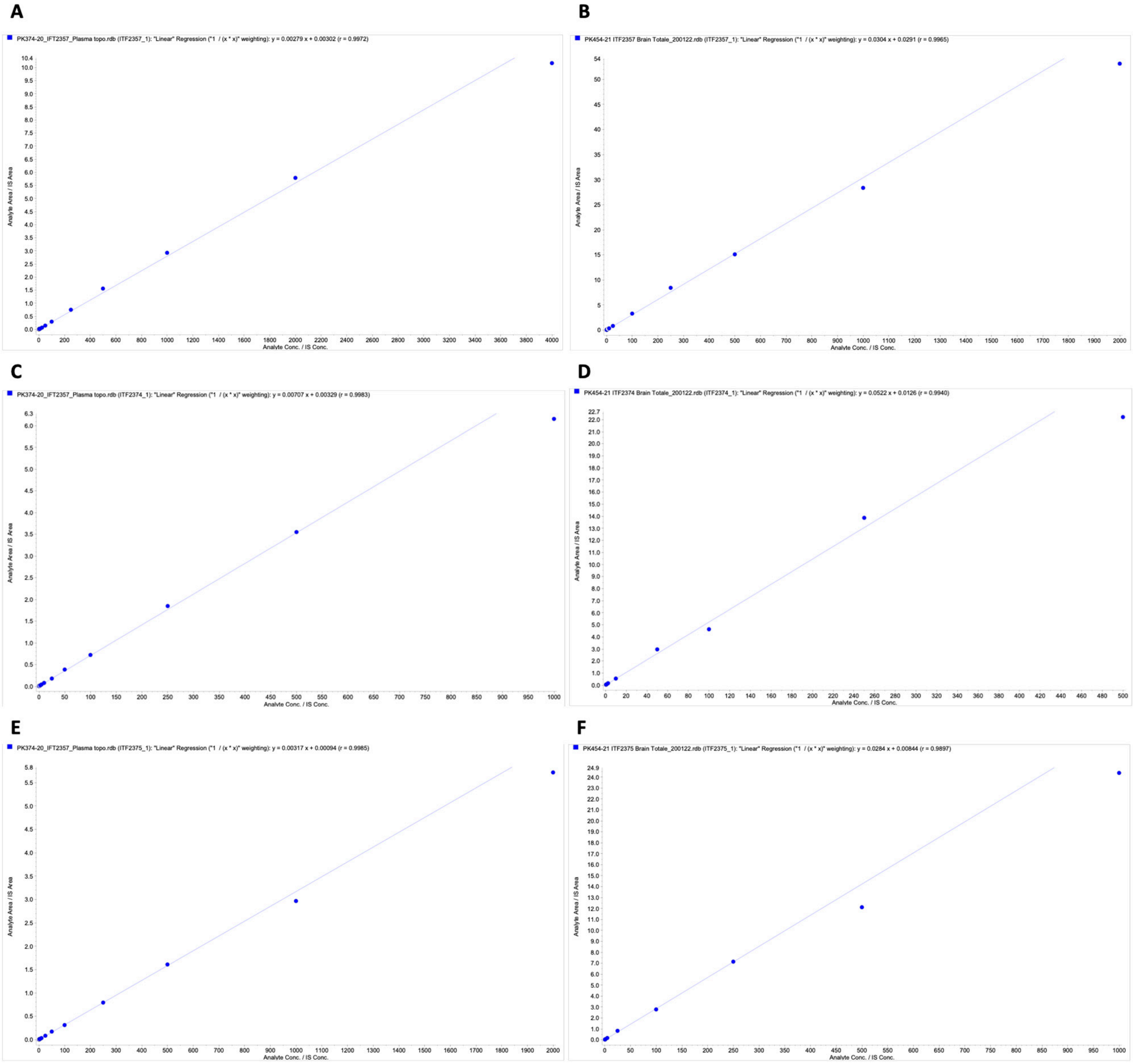

Figure S2. Panels **(A)** and **(B)**: Calibration curves for Givinostat in plasma (analytical range 2.5-4000 ng/mL) and brain homogenate (analytical range 0.5-2000 ng/mL)**.** Panels **(C)** and **(D)**: Calibration curves for ITF2374 plasma (analytical range 0.1-1000 ng/mL) and in brain homogenate (analytical range 0.5-500 ng/mL). Panels **(E)** and **(F)**: Calibration curve for ITF2375 in plasma (analytical range 0.5-1000 ng/mL) and in brain homogenate (analytical range 0.5-500 ng/mL).

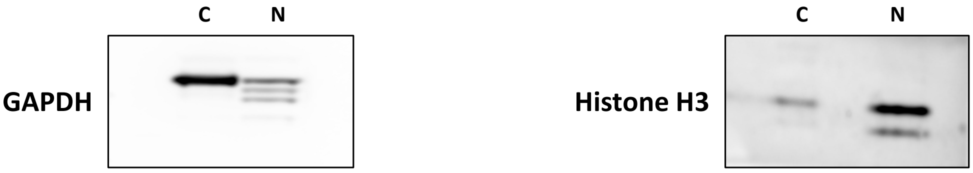

Figure S3. Reliability of cell fractions from Gli36ΔEGFR-2. The cytoplasm (C) and nucleus (N) were extracted after treatment with LIP/m-GIV using NE-PER^{TM} Nuclear and Cytoplasmatic Extraction Kit. Reliability of cell fractions were assessed by WB using GAPDH and Histone H3 primary antibodies. As expected, GAPDH protein was predominantly expressed in cytoplasm, while Histone H3 was expressed in nucleus. These WB are representative images of non-treated controls.

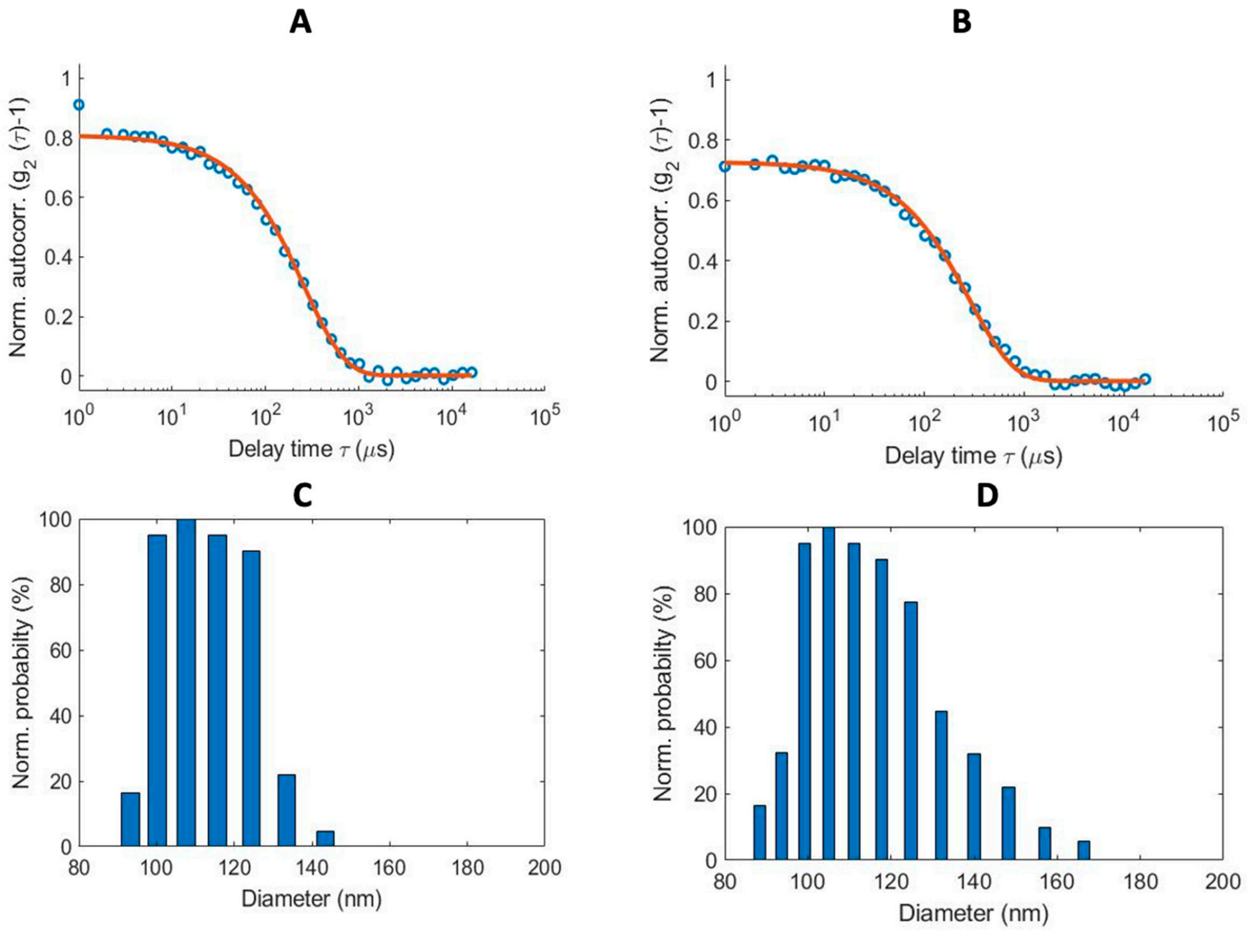

Figure S4. Representative autocorrelation functions with mono-exponential fit curve and CONTIN analysis of particle size distribution for LIP-GIV **(A-C)** and LIP/m-GIV **(B-D)** determined by DLS.

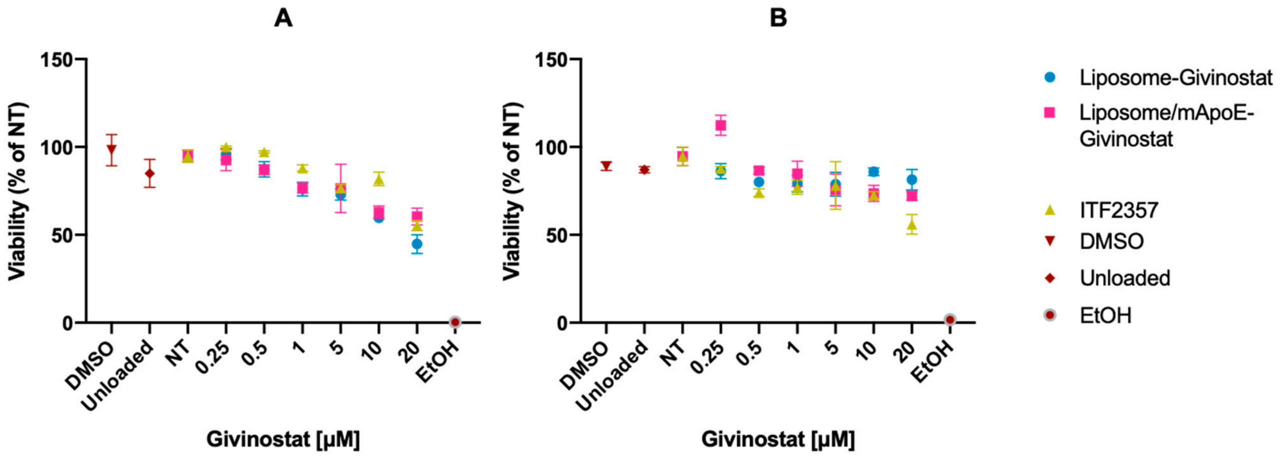

Figure S5. Evaluation of cytotoxicity on hCMEC **(A)** or Huvec **(B)** cell lines treated with LIP-GIV, LIP/m-GIV or free Givinostat for 24 h. "Unloaded" sample represents the unfunctionalized liposome without any drug loaded and it was used at the same lipid concentration as 20 µM dose (lipid concentration = 0.153 mM). Control DMSO was given in equivalent microliters than the highest dose of the inhibitor. Half-an-hour before the assay, three wells were pre-treated with 100% EtOH to provide near 100% mortality as a control. NT were established as controls at 100% viability. Each graph is the result of three independent experiments ± SD.

Table S2. Plasma levels of Givinostat and its metabolites

Group A (Free Givinostat)			Group B (LIP-GIV)			Group C (LIP/m-GIV)	
Time (h)	Givinostat (ng/mL)	SD	Time (h)	Givinostat (ng/mL)	SD	Givinostat (ng/mL)	SD
0.083333	1028	241.6	0.083333	23652	3759.4	21926	1200.6
0.166667	597	37.5	0.166667	22209	2328.9	19780	2863.8
0.25	431	109.4	0.25	21988	1554.3	19281	1298.4
0.5	256	78.3	0.5	22607	4154.0	17081	2659.6
1	75	26.9	2	18655	3806.8	13569	2429.7
2	19	3.8	6	14721	2639.0	15171	3661.8
4	6	2.5	24	1664	578.9	1095	1143.5
6	4	1.5	48	27	31.5	7	4.3
Time (h)	ITF2374 (ng/mL)	SD	Time (h)	ITF2374 (ng/mL)	SD	ITF2374 (ng/mL)	SD
0.083333	93.3	7.6	0.083333	85.7	5.9	66.4	4.4
0.166667	135.3	13.4	0.166667	82.1	3.9	70.7	16.8
0.25	126.4	12.6	0.25	85.2	8.0	60.7	11.6
0.5	143.7	15.2	0.5	64.5	4.2	52.5	14.0
1	109.9	33.0	2	153.9	22.9	148.6	35.1
2	55.3	13.7	6	278.6	141.2	343.3	39.4
4	38.4	5.5	24	25.0	30.9	24.2	30.0
6	18.2	8.5	48	<LOQ	-	<LOQ	-
Time (h)	ITF2375 (ng/mL)	SD	Time (h)	ITF2375 (ng/mL)	SD	ITF2375 (ng/mL)	SD
0.083333	860.4	101.7	0.083333	736.5	43.4	557.7	40.1
0.166667	1509.6	158.5	0.166667	580.4	39.7	516.8	72.6
0.25	1611.4	112.0	0.25	558.9	38.7	390.6	32.8

0.5	1398.8	148.5	0.5	402.0	42.9	328.2	62.7
1	615.1	39.2	2	336.2	69.1	290.2	20.1
2	231.1	33.9	6	415.8	58.3	551.0	101.3
4	81.0	34.5	24	35.7	38.8	29.8	32.6
6	24.8	15.0	48	1.8	0.9	1.3	0.7

n=3, if not stated otherwise.

Table S3. Brain levels of Givinostat and its metabolites

Group A (Free Givinostat)			Group B (LIP-GIV)			Group C (LIP/m-GIV)	
Time (h)	Givinostat (ng/g)	SD	Time (h)	Givinostat (ng/g)	SD	Givinostat (ng/g)	SD
0.083333	102	2	0.083333	455	46	621	75
0.166667	101	21	0.166667	463	137	625	90
0.25	90	10	0.25	399	53	674	65
0.5	83	19	0.5	406	89	489	424
1	44	4	2	279	28	456	56
2	30	4	6	212	40	322	27
4	13	2	24	11	10	15	5
6	7	3	48	<LOQ		<LOQ	
**Time (h)**	**ITF2374 (ng/g)**	**SD**	**Time (h)**	**ITF2374 (ng/g)**	**SD**	**ITF2374 (ng/g)**	**SD**
0.083333	33.1	1.4	0.083333	28.5	11.4	22.6	11.2
0.166667	51.0	3.6	0.166667	30.9	11.7	25.7	11.9
0.25	43.2	1.8	0.25	31.0	6.7	22.5	0.6
0.5	64.8	12.0	0.5	25.7	8.9	14.6	3.1
1	55.7	14.1	2	22.3	6.5	20.9	3.8
2	28.7	3.8	6	2.6	1.2	26.5	3.1
4	16.2	1.0	24	2.6	1.2	5	5
6	6.7	3.2	48	<LOQ	3.2	<LOQ	-
**Time (h)**	**ITF2375 (ng/g)**	**SD**	**Time (h)**	**ITF2375 (ng/g)**	**SD**	**ITF2375 (ng/g)**	**SD**
0.083333	13.6	2.0	0.083333	13.6	2.0	4.2	2.8
0.166667	27.7	4.7	0.166667	27.7	4.7	2.9	0.2
0.25	25.4	4.8	0.25	25.4	4.8	2.8	0.6
0.5	25.8	7.5	0.5	25.8	7.5	<LOQ	
1	9.9	1.2	2	9.9	1.2	<LOQ	
2	3.5*	-	6	<LOQ		<LOQ	
4	0.3*	-	24	<LOQ		<LOQ	
6	4.3*	0.4	48	<LOQ		<LOQ	

*n=3, if not stated otherwise. *n=1*

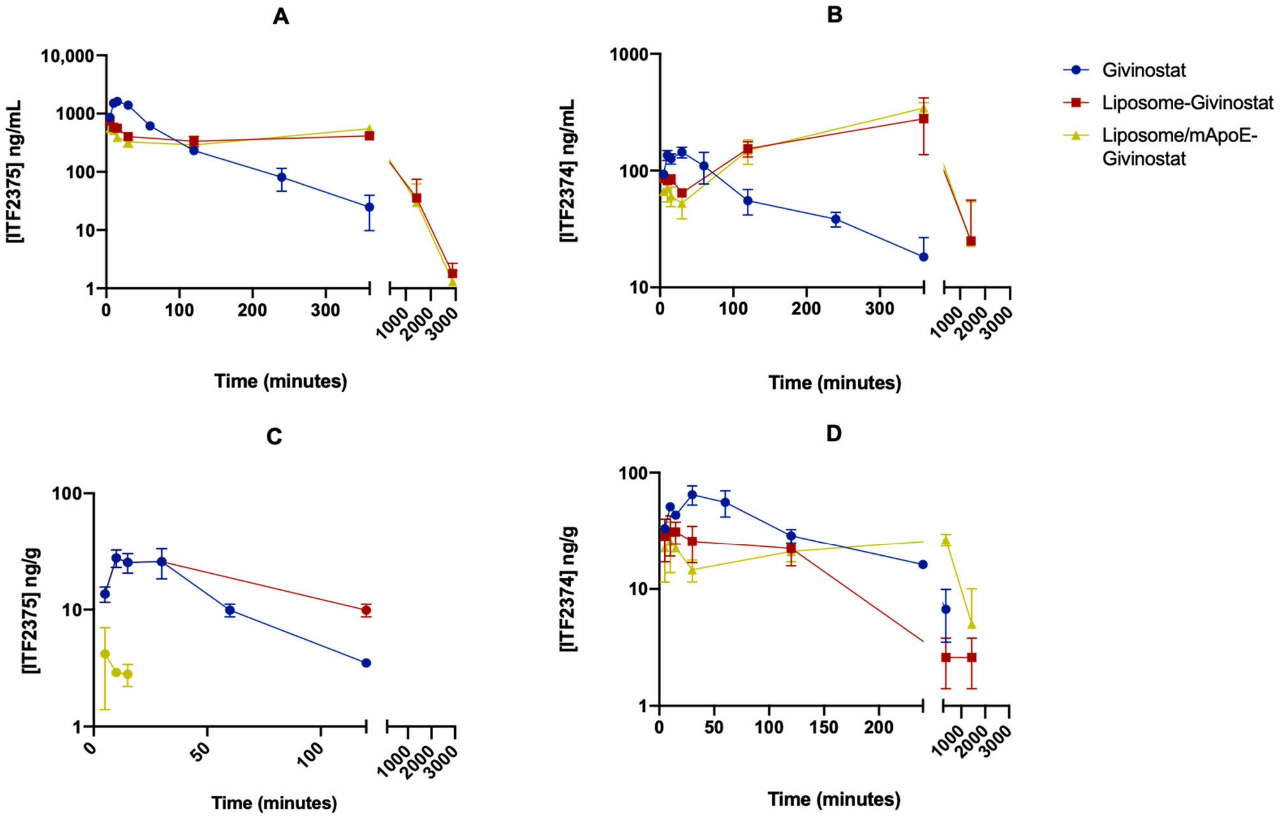

Figure S6. Distribution of Givinostat metabolites in brain and plasma after i.v. administration of free Givinostat, LIP-GIV or LIP/m-GIV. Panels **(A)** and **(B)** show the plasma distribution of ITF2375 and ITF2374 from 5 min to 48 h post-injection. Panels **(C)** and **(D)** show brain distribution of ITF2375 and ITF2374 from 5 min to 48 h post-injection. Data were obtained through LC-MS/MS method.

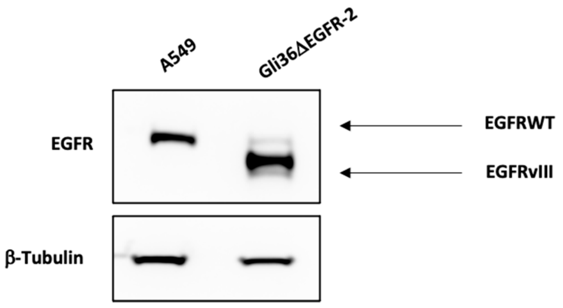

Figure S7. Expression of EGFR in A549 and Gli36ΔEGFR-2 cell lines. Protein levels were assessed by WB and equal loading was confirmed by β-tubulin antibody. EGFRWT (170 kDa) and EGFRvIII (140-155 kDa) are indicated by the arrows. Lung carcinoma A549 cell line was used as a representative control of a cell line carrying a WT expression of EGFR.

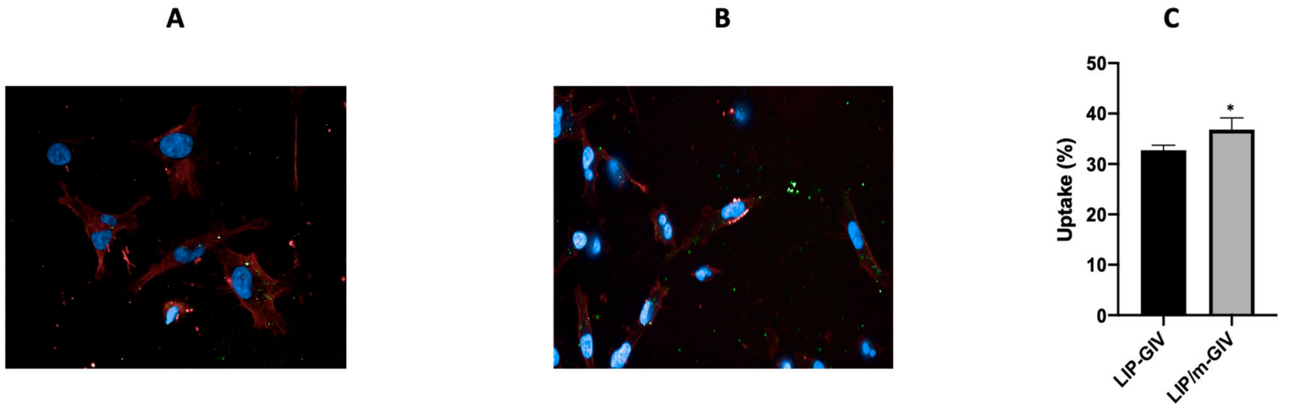

Figure S8. Targeting efficacy of fluorescent-labeled liposomes **(A)** and mApoE-liposomes **(B)** on Gli36ΔEGFR-2. Cells were stained with Hoechst (blue) and CellMask™ Deep Red Actin Tracking Stain (red). The liposomes fluorescence-associated is visible as green spots. Panel **(C)** shows the percentage of liposomes targeting efficacy. Results are presented as five independent experiments ± SD. *, $p < 0.05$, unpaired Student's t test.

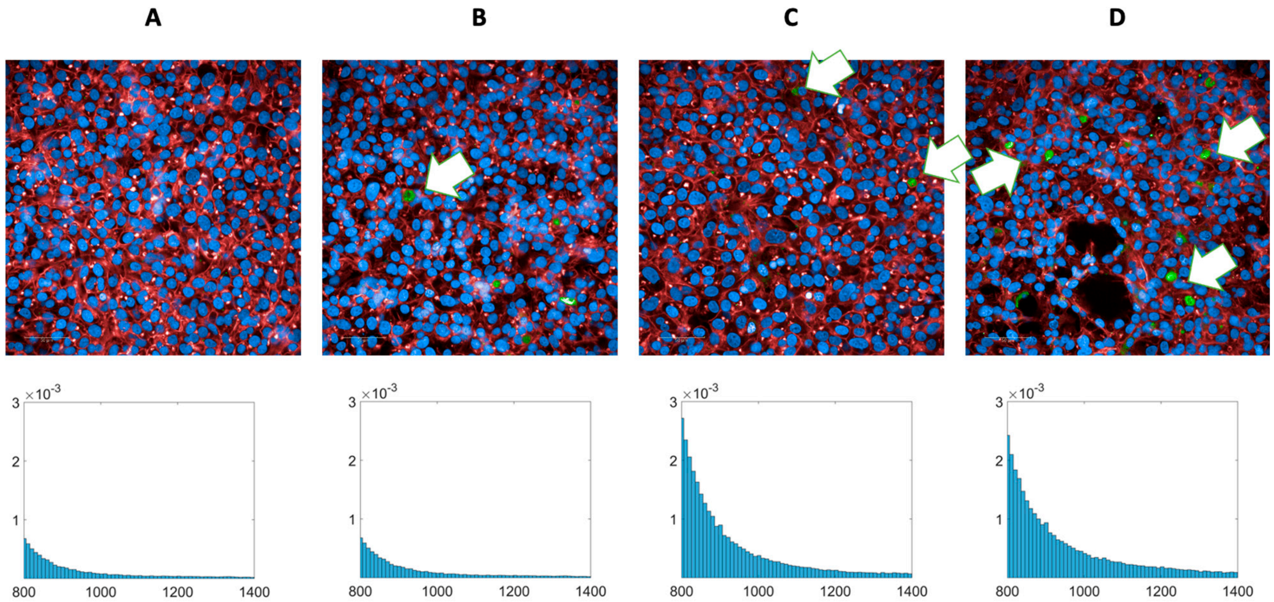

Figure S9. Increase of cleaved Caspase-3 in Gli36ΔEGFR-2 cells after treatment with Givinostat 0.25 μM **(B)**, 0.5 μM **(C)** or 1 μM **(D)**. **(A)** represents non-treated cells, used as a control. Cells were stained with Hoechst (blue), Phalloidin (red) and cleaved Caspase-3 (green). Immunofluorescence were performed using the Operetta CLS High Content Analysis System (Perkin Elmer). Arrows indicate green fluorescence. Results are presented as explicative images derived from two independent experiments. Quantitative measurements for Caspase-3 accumulation after treatment were presented as histograms showing intensities of the green channel images fixing the threshold >800 a.u.
